# Vibrational Dynamics in crystalline 4-(dimethylamino) benzaldehyde: Inelastic Neutron Scattering and Periodic DFT Study

**DOI:** 10.3390/ma15020475

**Published:** 2022-01-08

**Authors:** Mariela M. Nolasco, Paulo J. A. Ribeiro-Claro, Pedro D. Vaz

**Affiliations:** 1 CICECO, Departamento de Química, Universidade de Aveiro, P-3810-193 Aveiro, Portugal; prc@ua.pt; 2 Champalimaud Foundation, Champalimaud Centre for the Unknown, 1400-038 Lisboa, Portugal; pedro.vaz@fundacaochampalimaud.pt

**Keywords:** density functional theory, inelastic neutron scattering, vibrational assignment, molecular crystal, methyl torsion

## Abstract

The structure and dynamics of crystalline 4-(dimethylamino) benzaldehyde, 4DMAB, are assessed through INS spectroscopy combined with periodic DFT calculations. The excellent agreement between experimental and calculated spectra is the basis for a reliable assignment of INS bands. The external phonon modes of crystalline 4DMAB are quite well described by the simulated spectrum, as well as the modes involving low-frequency molecular vibrations. Crystal field splitting is predicted and observed for the modes assigned to the dimethylamino group. Concerning the torsional motion of methyl groups, four individual bands are identified and assigned to specific methyl groups in the asymmetric unit. The torsional frequencies of the four methyl groups in the asymmetric unit fall in a region of ca. 190 ± 20 cm^−1^, close to the range of values observed for methyl groups bonding to unsaturated carbon atoms. The hybridization state of the X atom in X-CH_3_ seems to play a key role in determining the methyl torsional frequency.

## 1. Introduction

Molecular interactions lie in a field that is still far from being fully understood. Assessing such molecular interactions unravels understanding of not only its structure but, more importantly, how a system’s dynamics contributes at the molecular level to the observable behavior at the macroscopic level. This can be done using a complete toolbox including crystallography (X-ray and neutron), nuclear magnetic resonance and vibrational spectroscopy (infrared and Raman). The latter offer extremely valuable information about the local structural environments of molecules. By using chemical bonds as probes, vibrational spectroscopy provides information about how a given molecule “communicates” with its surroundings. Such vibrational optical spectroscopy techniques can be complemented with the use of inelastic neutron scattering (INS). While IR and Raman are optical spectroscopy techniques relying on optical modes that are symmetry-allowed, INS does not obey the same selection rules, with the intensity of the bands being proportional the incoherent neutron scattering cross-section and corresponding atomic displacements in a given vibrational mode. In INS, the largest scattering cross-section is found for hydrogen, thus making this technique ideal to study organic or other hydrogenous systems. Furthermore, INS is of paramount importance since it makes it possible to study low-wavenumber (and high-amplitude) vibrational modes with high sensitivity, which provides critical structural information about molecular systems that cannot be accessed using IR or Raman.

The power of INS to study condensed systems is strongly and accurately complemented by the use of periodic density functional theory (periodic DFT) calculations in a cooperative fashion [1,2,3,4,5,6,7,8,9,10]. DFT methods, whether discrete or periodic, offer extreme and valuable assistance by predicting vibrational normal-mode eigenvectors. However, periodic methods offer unquestionable further advantage over their discrete counterparts by predicting those vibrational modes found in crystalline systems, such as crystal field splitting and molecular librational and translational modes, which are observed experimentally with INS. Therefore, the combination of INS with periodic DFT methods yields a very accurate yet convenient systematic approach to test molecular models and explain molecular interaction phenomena.

In this work, we present a study where the INS spectrum of 4-(dimethylamino) benzaldehyde (4DMAB) is assessed, and its insights are supported by periodic DFT calculations. 4DMAB closes a sequence of benzaldehyde derivatives, the INS spectra of which were obtained prior to the determination of their crystal structure (within a related project with C-H^…^O hydrogen bonds) and are now being used to assess the description of vibrational spectra from combining INS spectroscopy with periodic DFT calculations [4,9,10]. In the INS spectrum of 4DMAB, the low-wavenumber region (<300 cm^−1^) is dominated by modes arising from the substituent groups of the phenyl ring, namely benzaldehyde, amine and its methyl group moieties beyond the translational and librational modes. The presence of band splitting and the dynamics of methyl torsions in the crystal were confirmed both experimentally and computationally and are presented and discussed.

## 2. Materials and Methods

4-(dimethylamino) benzaldehyde was obtained commercially at high-purity grade (Sigma-Aldrich, CAS number 100-10-7, 99% purity, St. Louis, MO, USA) and used without further purification.

Inelastic neutron scattering spectroscopy: The INS spectrum was obtained using an indirect geometry time-of-flight spectrometer, TOSCA, at the ISIS Neutron and Muon Source at the Rutherford Appleton Laboratory (Chilton, UK) [11,12]. The sample of 4-(dimethylamino) benzaldehyde, with a total amount of ca. 2 g, was loaded into a flat, thin-walled aluminium can of 4.8 cm height × 4 cm width × 2 mm path length, sealed with an indium wire and mounted perpendicularly to the beam by using a regular TOSCA center stick. The INS spectrum, measured for the 16 to 8000 cm^−1^ energy-transfer range, was recorded below 15 K with a resolution of ∆E/E ≈ 1.5%. MANTID program (version 4.0.0) [13] was used to convert the data to the to the conventional scattering law, S(Q,ν) vs. energy transfer (in cm^−1^).

Density functional theory (DFT) calculations: Discrete calculations on the structure of 4-(dimethylamino) benzaldehyde molecule were performed at the Perdew-Burke-Ernzerhof (PBE) [14] level of theory with the 6–311G(d,p) basis set using the Gaussian 09w program version [15]. Frequency calculations ensured that the optimized geometries were real minima (no negative eigenvalues) and provided the infrared and Raman intensities. The “OPT = ModRed” option of G09, by using a step size of 10° to scan the relevant CNCH dihedral angle, allowed the potential energy functions for internal rotations to be obtained.

Periodic DFT calculations were performed using the plane-wave/pseudopotential method as implemented in the CASTEP code [16,17].The exchange-correlation energy in the calculation was described by the PBE functional [14]. The plane-wave cut-off energy of 830 eV was taken with the 8 × 4 × 4 Monkhorst-Pack grid for the electronic sampling of the Brillouin zone (BZ). An essential prerequisite for lattice dynamics calculations is the equilibrium structure, which was obtained by LBFGS geometry optimization, after which the residual forces were converged to zero within 0.005 eV·A^−1^. From the reported crystal structure (CSD entry: ZZZMFY01) [18], the initial structure was taken and optimized, keeping constant the cell parameters. When using standard GGA functions, this is an important issue, as dispersion/van der Waals interactions description is defective, leading to unrealistic cell dimensions, as discussed elsewhere [19].

The diagonalization of dynamical matrices computed by the density-functional perturbation theory [20] with a coarse q-point grid of 4 × 2 × 1, accounting for 12 q-points to sample the BZ, allowed for the calculation of phonon frequencies. Additionally, to the direct evaluation of frequencies and intensities at the Γ-point, the phonon dispersion was evaluated along a high-symmetry path throughout the BZ.

The atomic displacements for each normal mode (obtained from the CASTEP and G09 outputs) were used by the program Jmol [21] to visualize the modes, as well as to generate the INS spectrum through the program AbINS [22]. AbINS, an open-source package implemented as a plugin to the neutron data-analysis software Mantid [13] accounts for the neutron-scattering cross sections, overtones and combination modes. All that information, together with instrument-specific E-Q windows, produced a calculated INS spectrum that is easily compared with the experiment. For all the calculated spectra shown, it should be emphasized that the transition energies were not scaled.

## 3. Results and Discussion

### 3.1. Molecular Geometry and Intermolecular Interactions

**Figure 1:**
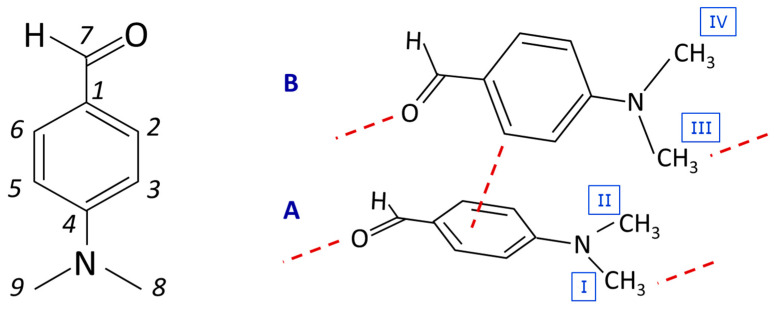
Representation of 4-(dimethylamino) benzaldehyde with the atom labelling used throughout the text (**left**) and a schematic representation of the crystal structure, evidencing the relevant intermolecular contacts present in the crystal (**right**). Molecules in the asymmetric unit are labelled A and B, and methyl groups are labelled I-IV.

Figure 1, left, presents the molecular structure of 4-(dimethylamino) benzaldehyde (4DMAB), along with the numbering scheme adopted in this work. The crystal structure of 4DMAB (monoclinic, space group P21/n, Z = 8) has been reported by Gao and Zhu [18] and by Vicente et al. [23]. Gao and Zhu [18] state that 4DMAB “*crystallizes with two independent but essentially identical molecules in the asymmetric unit, which are linked via a C-H^…^*π *interaction. In both molecules, the aldehyde and dimethylamine groups are essentially coplanar with the attached benzene ring. In the crystal structure, C-H^…^O hydrogen bonds link one type of independent molecules into a chain along the a axis. In addition, the structure is stabilized by* π*-stacking interactions involving the benzene rings.*”. The two molecules in the asymmetric unit are schematically represented in Figure 1, right, evidencing the C-H^…^π and C-H^…^O contacts (along with the labels A and B for the two molecules and I-IV for the methyl groups).

A comparison between some selected geometric parameters obtained from X-ray [18], periodic calculations (CASTEP, see Experimental details) and discrete single-molecule calculations (G09, see Experimental details) can be found in Table 1. Overall, comparing the experimental and calculated crystal structures (X-ray vs. CASTEP), there is a good agreement. Deviations below 5% and 2% RMS are observed for the coordinates of all atoms (excluding hydrogen atoms) and for the bond lengths (excluding C-H bonds), respectively. However, the presence of some well-known limitations of PBE calculations on CASTEP geometric parameters is verified. An overestimation of the C=O bond length is clearly observed, leading to underestimation of the C=O stretching mode, already discussed elsewhere [19,24], and the pi-stacking distance evidences the foresee bias to large values. If not constrained by cell dimensions [19], this distance would deviate to physically meaningless values.

Geometry obtained from single molecule (discrete) calculations (G09) at the PBE/6-311G(d,p) level is in line with the one recently reported at the B3LYP/6-31G(d,p) level [25], and no large deviations from the CASTEP geometry are observed. For the isolated molecule, all non-hydrogen atoms are in the same molecular plane, with both substituents being coplanar with the aromatic ring. The dihedral angles shown in Table 1 are somewhat deviated from planarity, an effect of crystal packing already observed in similar systems [4,9,10].

### 3.2. Calculated vs. Experimental Spectra

**Figure 2:**
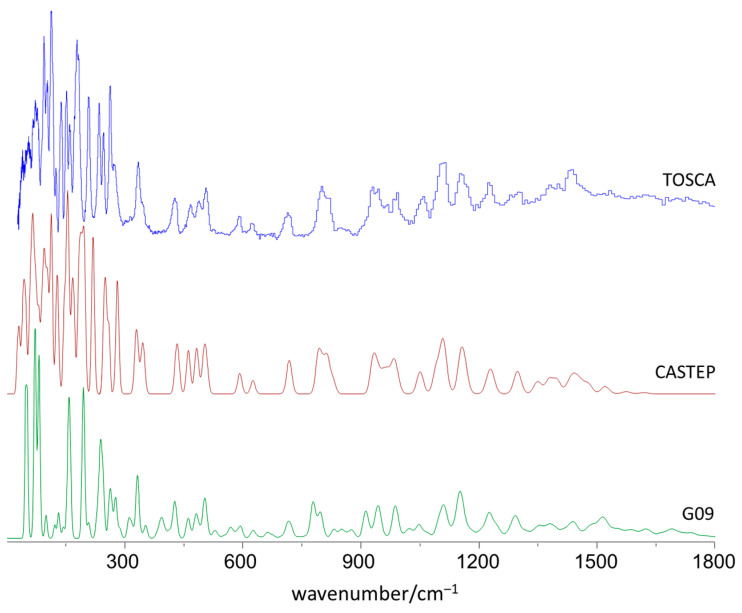
The INS spectra of 4-(dimethylamino) benzaldehyde in the 25–1800 cm^−1^ range: experimental (**top**), simulated from periodic calculations (**middle**) and from single-molecule discrete calculations (**bottom**).

**Figure 3:**
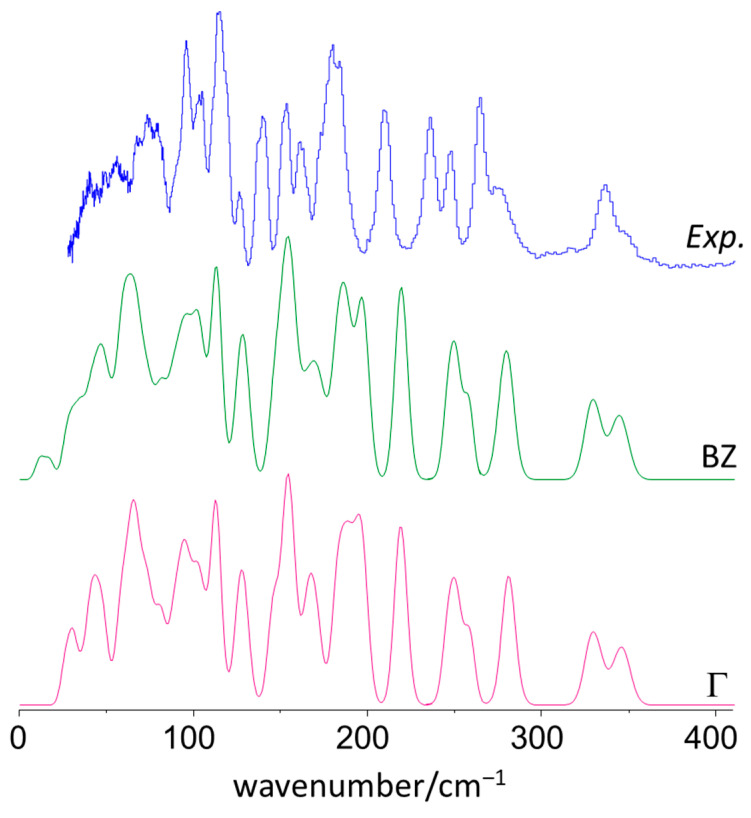
Experimental spectrum of 4-(dimethylamino) benzaldehyde in the range below 300 cm^−1^ (**top**) compared with the corresponding INS spectra calculated at the Γ-point (Γ, **bottom**) and averaged by the dispersion throughout the Brillouin zone (BZ, **middle**).

Figure 2 compares the experimental INS spectrum of 4DMAB (TOSCA) with the simulated INS spectra obtained from periodic (CASTEP) and discrete (G09) calculations, up to 1800 cm^−1^. Figure 3 presents a detailed view for the low-wavenumber region and its description without and with considering the effect of phonon dispersion (simulated spectra from Γ-point and with sampling over the Brillouin zone, respectively).

These figures evidence the excellent agreement between the experimental spectrum and the simulated spectrum derived from the periodic approach, in line with the results previously reported for similar benzaldehyde derivatives [4,9,10]. Even for the low-wavenumber modes (Figure 3), a region for which the computational description has been found to be more demanding [10,26], the agreement between calculated and experimental profiles is very satisfactory. This agreement supports the reliable assignment of the vibrational modes of crystalline 4DMAB, as discussed below.

Concerning the simulated spectrum from the discrete model (Figure 2, bottom), a reasonable agreement with the experimental spectrum is observed but only from ca. 700 cm^−1^ upwards, evidencing the difficulties of the single molecule approach to describe the crystalline structure. In the region below ca. 700 cm^−1^, the agreement is clearly unsatisfactory in both the position and intensity of the calculated bands. Of course, in the region where the contributions from collective modes are expected to prevail (ca. <100 cm^−1^), the calculated spectrum for a single molecule is meaningless.

The crystallographic unit cell of 4DMAB (22 atoms per isolated molecule) contains 176 atoms. A total of 525 optical phonon modes are expected: 480 vibrational modes from the eight molecules (120 Ag + 120 Au + 120 Bg + 120 Bu) and 45 external modes describing translations and rotations (21 translational modes plus 24 librational modes, 12 Ag + 11 Au + 11 Bg + 11 Bu).

From the 60 normal vibrational modes of each individual molecule, 30 are related to the phenyl ring, six to the aldehyde group and the remaining 24 to the dimethylamine fragment. The ring-related modes can be described as 12 stretching modes (6 × νCC, 4 × νCH and 2 × νC–R; R = substituents, CHO/N(CH_3_)_2_), nine in-plane deformation modes (3 × α ring, 4 × βCH and 2 × βC–R) and nine out-of-plane deformation modes (3 × δ ring, 4 × γCH and 2 × γC–R). The six modes related to the aldehyde group can be described as two stretching modes (νC=O, νC-H), two in-plane deformation modes (βC=O, βC-H), and two out-of-plane deformation modes (γC=O, γC-H), one of which is, in fact, the torsional mode (τC–CHO). The 24 modes related to the dimethylamine group can be described in terms of six NC2 modes (analogous to the 6 CHO modes) and of the well-known 2 × 9 –CH_3_ modes (three stretching modes, five deformation modes and one torsional mode for each methyl group).

In INS, due to the absence of selection rules, all vibrational modes are permitted. However, since the signal intensity is highly dependent on the motions of hydrogen atoms (hydrogen atoms present the largest scattering cross-section and the largest displacements during vibration), only some modes have measurable intensity. This includes the low-frequency/high-amplitude vibrational modes, an advantage of INS relative to its optical counterparts. Table 2 presents the observed and calculated band maxima and their approximate descriptions in terms of molecular vibrations. The “approximate description” of the normal modes refers to the most relevant contribution to each normal mode, despite their mixed nature resulting from coupling between oscillators. This description is assumed to be the most useful alternative to the more rigorous but often cumbersome description based on the potential energy distribution (PED), as discussed elsewhere [19].

**Figure 4:**
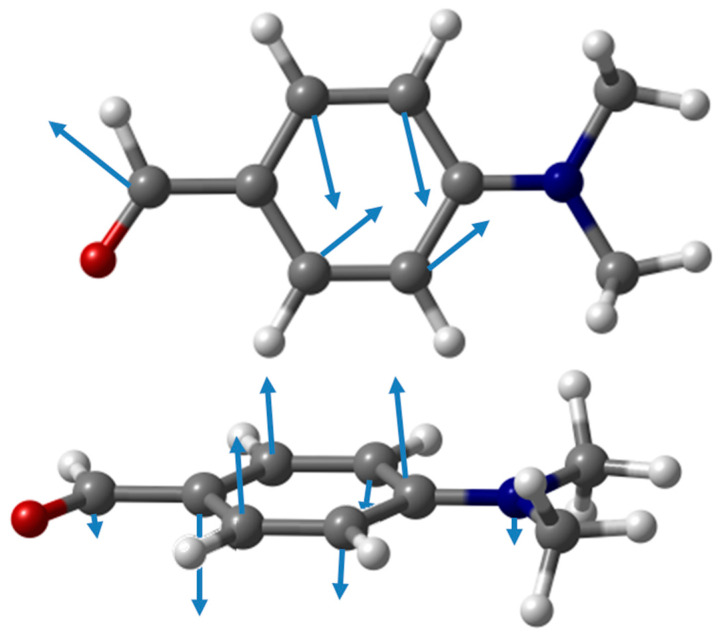
Atomic displacements of heavy atoms in one of the phonon modes contributing to the band at ca. 719 cm^−1^. The large displacements of hydrogen atoms were omitted for clarity.

Apart from the external or collective modes, phonon modes in 4DMAB crystal were found to be grouped in combinations of eight identical molecular normal modes—generally, combinations of four A-type molecules and four B-type molecules—giving rise to the observed INS band maxima. In that sense, bands in Table 2 are described in terms of molecular normal modes of vibration. However, since 4DMAB crystal results from the packing of molecules contained in nearly orthogonal planes (namely, molecules linked via a C-H^…^π interaction), mixing between in-plane and out-of-plane molecular modes is observed. In a few cases, the phonon mode includes an in-plane mode of one B-type molecule and an out-of-plane mode of the neighboring A-type molecule. Figure 4 illustrates the situation for one of the phonon modes contributing to the 719 cm^−1^ band, which is nominally described as “ring out-of-plane deformation” but includes contributions from the “ring in-plane deformation” of the B-type molecules. Since the molecules are in orthogonal planes, all the atoms in both molecules move along the same “direction” (more rigorously, move in parallel planes).

A straightforward validation of the model is provided by the excellent description of the INS spectrum obtained from the periodic calculations, thus allowing a trustful assignment of vibrational modes of crystalline 4DMAB. The vibrational assignment of IR and Raman spectra of 4DMAB has been reported, based on an empirical force-field normal coordinate analysis [27] and on discrete calculations at the B3LYP/6-31G(d,p) level [15]. A partial assignment was also reported by the authors in an attempt to describe the spectrum of the crystalline samples from the sum of dimer contributions [28].

There is a general concordance among assignments in the medium-wavenumber region, with a few discrepancies concerning the substituent groups. For the most relevant discrepancies, which occur for the low-wavenumber region, the combination of periodic calculations and INS spectrum provides clarification, as discussed below.

For instance, Rocha et al. [25] relate the 177 cm^−1^ band in the Far-IR spectrum to the torsion of the –CHO group, a mode assigned to the Far-IR band at ca. 109 cm^−1^ by Kushto and Jagodzinski [27]. Rocha et al. also report and assign a Far-IR band at 247 cm^−1^ to the in-plane bending motion of the dimethylamino substituent, a band not identified by Kushto and Jagodzinski in their work. According to CASTEP calculations (Table 2), the torsional –CHO mode is better assigned to the INS band at 105 cm^−1^, in agreement with Kushto and Jagodzinski, and the band at 247 cm^−1^ is better related with the out-of-plane bending motion of the dimethylamino substituent. However, since both works ignore the intermolecular interactions in the crystal and Kushto and Jagodzinski did not include the internal rotations of the methyl groups and the dimethylamino group in their calculations, their analysis of the low-wavenumber region is necessarily incomplete and defective.

One of the most evident effects of the intermolecular interactions due to crystal packing is the factor group splitting. In fact, the factor group splitting for a few modes in 4DAMB crystal observed in the INS spectra is foreseen by periodic calculations. Factor group splitting, as would be expected, is present in a crystal cell with eight molecules (eight molecular modes are expected to combine into eight phonon modes), as its magnitude is influenced by the magnitude of the intermolecular forces in the crystal. For most of the vibrational modes, the factor-group components are not separated (considering the spectral resolution) and do not influence the observed spectra.

As can be seen in Table 2, crystal splitting is predicted to have observable magnitude for the phonon modes below 350 cm^−1^ of 4DMAB but only in the modes related to the dimethylamino group. In fact, none of the aldehyde-related modes (C-CHO out-of-plane bend, in-plane bend, and torsion) presents measurable splitting. This is a particular feature of 4DMAB since for the other previously reported 4-substituted benzaldehyde crystals [4,9,10], factor group splitting was observed for the –CHO modes (at least for the –CHO torsional mode).

On the other hand, the splitting is clearly predicted and observed for the corresponding –N(CH_3_)_2_ modes, plus the CH_3_ torsional modes. The notable exception is the in-plane bending of the C-N(CH_3_)_2_, for which a possible splitting is observed and assigned, as observed in Table 2, although it is not predicted from theoretical calculations. For this pair of bands, 265 and 275 cm^−1^, the alternative assignment of a single band plus a 0–2 transition cannot be excluded.

**Figure 5:**
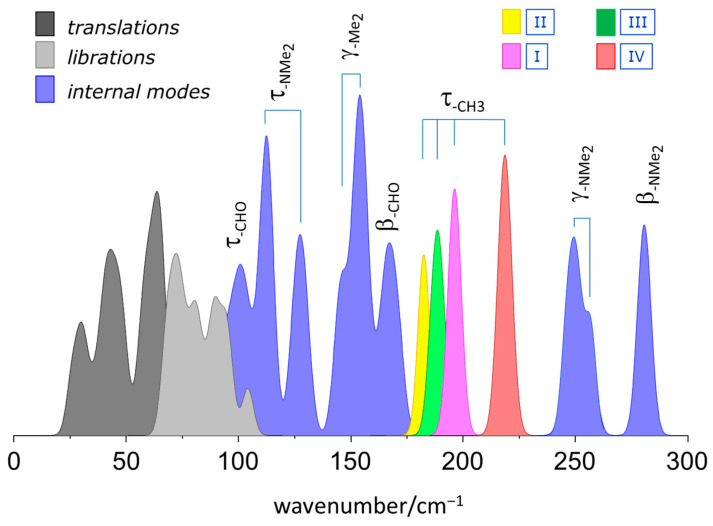
Pictorial description of the calculated band maxima in the low-wavenumber region (from periodic-DFT calculations). External modes (translations and librations) in grey, internal modes in blue, except for the CH_3_ torsional modes, with a differentiated color code. The CH_3_ torsional modes are labelled I–IV, according to Figure 1.

Figure 5 illustrates the phonon distribution in the calculated INS below 300 cm^−1^, with special emphasis on the bands ascribed to methyl torsional motions. Each torsional mode is labelled according to its position, as detailed in Figure 1. The labels I–IV distinguish the methyl groups according to their positions in the asymmetric unit. The methyl groups are all inequivalent due to their neighboring contacts (as can be seen in Figure 1). For instance, methyl group I is engaged in C-H^…^O bonding, while methyl group II is not. Identically, methyl group III presents a shorter C-H^…^O contact than methyl group IV, which, in turn, is closer to the π-system.

The methyl torsion with the lowest wavenumber is II, while that with the highest energy is III. Torsional modes I and II are highly localized in the corresponding methyl group, but modes III and IV are less localized and can alternatively be described in terms of (III + IV) symmetrical and asymmetrical combinations, respectively. The predicted frequencies and intensities of the methyl torsion modes are in very good agreement with the experimental frequencies and intensities, which suggests that a classical approach is enough to describe the behavior of methyl torsion in this system.

The torsional frequencies of the four methyl groups fall in a region of ca. 190 ± 20 cm^−1^. According to the literature, torsional modes of methyl groups bonded to sp^2^ carbon atoms appear at ≤200 cm^−1^, while those bonded to sp^3^ carbon atoms occur at ~250 cm^−1^ [29]. For methoxybenzaldehydes [10], it was found that methyl groups bonded to (sp^3^) oxygen atoms also occur at ~250 cm^−1^, thus pointing to a similar nature of the potential energy barrier for the rotation along the C(sp^3^)-CH_3_ and O-CH_3_ bonds. The present work places the N-CH_3_ torsional motion close to the C(sp^2^)-CH_3_ case, which may suggest a different tendency between oxygen and nitrogen atoms. It should be mentioned, however, that the planarity of 4DMAB can be ascribed to a resonance structure with an sp^2^ nitrogen atom. To the best of our knowledge, the data for methyl torsional motions in non-aromatic amines are scarce, but in the case of the quaternary ammonium ion choline, with methyl groups bound to an sp^3^ nitrogen atom, torsional modes in the range of 250–330 cm^−1^ have been observed in the INS spectrum [30]. On the whole, these observations point to the importance of hybridization of the atom to which methyl group is bound as the determining factor for the torsional barrier of the methyl groups.

For the isolated molecules, there is no significant difference between the two methyl groups, and the potential energy scan for the rotation of a single methyl group yields a V_3_ potential of ca. 430 cm^−1^ (at PBE/6-311G** level). This is considerably lower than the values reported for N-methylaniline, obtained from similar discrete DFT calculations (ca. 760 cm^−1^) [31] or derived from microwave spectroscopy (975 cm^−1^) [32]. The planarity of the N(CH_3_)_2_ moiety and the presence of a second methyl group are expected to contribute to this difference in barrier-energy heights.

## 4. Conclusions

INS spectroscopy offers unique access to the low-wavenumber modes, particularly those involving large atomic displacements of hydrogen atoms, and has a synergic combination with periodic DFT calculations. All the gathered information in this work provides a good basis for interpreting the vibrational spectra and assessing the structure and dynamics of 4DMAB in the crystal form, given the excellent agreement between calculated and experimental INS spectra.

The external phonon modes of crystalline 4DMAB are quite well described by the simulated spectrum, a somewhat unusual situation, due to de limitations of periodic calculations (in short, low-wavenumber modes are known to collect calculations errors). In what concerns the dynamics of the low-wavenumber/high-amplitude molecular motions, the behavior of the –CHO and –N(CH_3_)_2_ substituents is dissimilar. Crystal field splitting is only observed for the modes assigned to the dimethylamino group and not for the aldehyde-related modes. It should be emphasized that the use of a simplified approximate description of the normal modes is not intended to hide the complex nature of some vibrational motions. Actually, in most of the low-wavenumber vibrations involving the substituent groups, the single-oscillator mode is more an exception than a rule. Hence, the description “–CHO modes” or “–N(CH_3_)_2_ modes” only identifies a dominant contribution to a complex vibrational mode.

The torsional frequencies of the four methyl groups fall in a region of ca. 190 ± 20 cm^−1^, close to the range of values observed for methyl groups bond to unsaturated carbon atoms. Comparing the INS data gathered for C-CH_3_, O-CH_3_ and N-CH_3_, the previously formulated general rule concerning torsional frequency of methyl groups [29] may be upgraded to: “torsional modes of methyl groups bonded to sp^2^ atoms occur at ≤200 cm^−1^, while those bonded to sp^3^ atoms appear at ~250 cm^−1^”.

## Figures and Tables

**Table 1:** Selected geometrical parameters of 4-(dimethylamino) benzaldehyde. Molecules in the asymmetric unit are labelled A and B, according to Figure 1.

	X-ray (A)	X-ray (B)	CASTEP (A)	CASTEP (B)	G09
Bond length/pm					
C7=O	121.2	120.4	123.7	123.7	122.5
C1-C7	145.4	145.8	144.9	145.3	146.8
C4-N	136.6	136.6	137.3	137.0	137.6
Bond angle/°					
C1-C7-O	125.1	126.2	126.10	125.7	125.4
C4-N-C8	121.2	120.9	120.7	120.9	120.4
Dihedral angle/°					
C2C1-C7O	1.4	1.6	1.5	0.3	0.0
C3C4-NC8	−2.4	4.7	−3.2	5.8	0.0
Distance/pm					
C8(H)^…^O	359.1	363.4	356.8	366.6	-
C2(H)^…^Cg ^1^		359.3		363.1	-
π...π (C1^…^C4′) ^2^	370.4		377.0		-

^1^ Distance between C2 and the center of aromatic ring of the neighboring molecule; ^2^ distance between C1 and C4 atoms of the π-stacking molecules (A).

**Table 2:** Experimental and calculated INS maxima for 4-(dimethylamino) benzaldehyde with vibrational mode assignments.

Calculated (CASTEP) ^1^	Experimental (TOSCA)	Approximate Description
1479	1473	βCH_3_
1442	1443	βCH_3_
1394	1408	βCH_3_
1382	1388	βCH(=O)
1298	1309	βCH
1229	1234	νC-CHO
1156	1161	βCH
1108	1116	βCH
1089	1116	rock CH_3_
1050	1065	rock CH_3_
985	993	γCH(=O)
963	970	γCH
940	951	γCH
930	933	νN-(CH_3_)_2_
814	825	γCH
795	806	γCH
718	719	δ ring
625	630	α ring
593	594	α ring
505	509	δ ring
483	490	β N-(CH_3_)_2_
461	470	β N-(CH_3_)_2_
433	431	δ ring
345	348	α ring
329	336	γ ring-CHO
-	275	(*see text*)
281	265	β ring-N(CH_3_)_2_
255	247	γ ring-N(CH_3_)_2_
251	237	
219	210	τ N-CH3
195	184	
189	180	τ N-CH_3_
182	172	
168	162	β ring-CHO
155	153	γ N-(CH_3_)_2_
146	138	
128	128	τ C-N(CH_3_)_2_
113	115	
101	105	τ C-CHO
95	90	Libration
83	75	Libration
66	67	Libration
49	51	Translation
43	39	Translation

^1^ Maxima in the INS simulated spectrum. ν, α, β, γ and τ stand for stretching, in-plane deformation, out-of-plane deformation and torsion modes, respectively.

## Data Availability

Data from the neutron scattering experiments can be accessed freely at https://data.isis.stfc.ac.uk/#/browse/facility/ISIS/instrument/17/facilityCycle/23/investigation/14824759/dataset (accessed on 6 November 2021).
